# A case report of idiopathic Hemophagocytic lymphohistiocytosis in an immunocompetent adult

**DOI:** 10.1002/ccr3.4006

**Published:** 2021-03-28

**Authors:** Tiago Rabadão, Leonor Naia, Filipa Ferreira, Mariana Teixeira, Marcelo Aveiro, Margarida Eulálio, Fernando Silva

**Affiliations:** ^1^ Internal Medicine Department Centro Hospitalar do Baixo Vouga Aveiro Portugal; ^2^ Hematology Department Centro Hospitalar do Baixo Vouga Aveiro Portugal

**Keywords:** fever of unknown origin, Hemophagocytic lymphohistiocytosis, hyperferritinemia, idiopathic, index of suspicion, sepsis‐like

## Abstract

Hemophagocytic lymphohistiocytosis poses a diagnostic dilemma due to the absence of specific clinical and laboratory findings, especially in adults. Despite greater recognition of the disease, secondary idiopathic forms are still reported.

## Graphical abstract

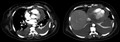

## 1 INTRODUCTION

The authors report the clinical case of an idiopathic Hemophagocytic lymphohistiocytosis (HLH) in an adult patient, first appearing with nonspecific symptoms, followed by sepsis‐like evolution requiring advanced life support. Despite exhaustive study, no etiology for HLH was identified, including infectious, malignancy, autoimmune, or genetic causes.

Hemophagocytic lymphohistiocytosis (HLH) is a severe inflammatory syndrome of excessive immune activation, due to cytokine overproduction.^1^, ^2^, ^3^ It is largely underdiagnosed in adults^4^ and is a life‐threatening disease, especially if diagnosed late. There are two forms of the disease: primary (that includes familiar forms) and secondary or acquired, usually due to infections, malignancy, and autoimmune/autoinflammatory diseases^1^, ^2^, ^3^; both forms have similar clinical characteristics.^4^ Despite the etiology, HLH is an important cause of fever of unknown origin and extreme elevation of ferritin, associated with multiple organ involvement. Initial signs and symptoms of HLH can mimic common diseases, like infections.^2^ Previously established diagnostic criteria (*HLH‐2004*) are controversial and do not reflect, especially in adults, all described clinical manifestations, due to extrapolation of pediatric recommendations and studies.^1^, ^2^, ^3^, ^4^ A new tool, *HScore*, was created to improve diagnosis, but clinical disease heterogeneity is not always represented by available scores. There is still a poor recognition of HLH but, nowadays, diagnosis of this clinical entity reemerged, motivated by COVID‐19 pandemic—an HLH‐like syndrome has been reported in association with SARS‐CoV‐2^5^ (this case was not contemporary to this situation).

## 2 CASE PRESENTATION

**FIGURE 1:**
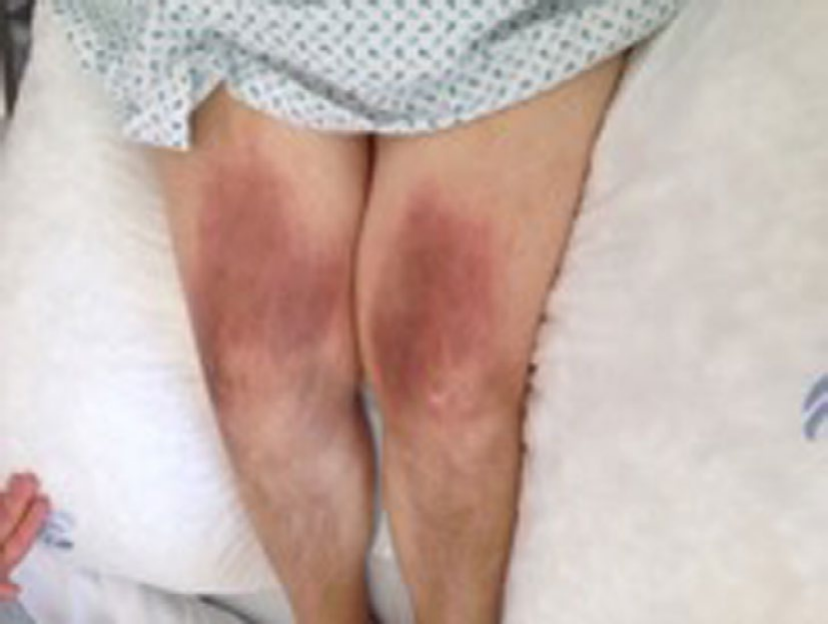
Scratchy skin lesions in lower limbs, present on patient admission

A 49‐year‐old woman presented with one‐week history of anorexia, asthenia, pruritus, abdominal pain, nausea, and liquid stools. The patient had poor living conditions and suffered from mild cognitive impairment and blindness since childhood. On admission, she presented with fever (38.2ºC), pain on right superior abdominal quadrant, and scratchy skin lesions (Figure 1); a short stature and a dysmorphic face were noted; no neurologic findings.

Laboratory study carried out showed: anemia, leukocytosis, and thrombocytosis; high sedimentation velocity, d‐dimers, fibrinogen, ferritin, lactate dehydrogenase (LDH), C‐reactive protein (CRP), triglycerides, soluble IL‐2 receptor, and interleukin‐6 levels; hypergammaglobulinemia; mild cythocolestasis; hypoalbuminemia; and low high‐density lipoprotein (HDL) values. Natural killer (NK) cell activity was normal (Table 1).

**TABLE 1:** Laboratory tests significant results

Parameters	Value (range)
Hemoglobin (g/dL)	**7,4** (11.5‐16.5)
Leukocytes (×10^9^/L)	**11** (4.1‐10.8)
Platelets (×10^9^/L)	**633** (150‐500)
Blood smear	*Rouleaux*, microcytosis, hypochromia, gross granulations and vacuols in neutrophil cytoplasma.
Ferritin (ng/mL)	**18953** (20‐291)
Sedimentation velocity (mm/h)	**130** (<20)
d‐dimers (ng/dL)	**18898** (<500)
Fibrinogen (mg/dL)	**502** (200‐393)
Sodium (mEq/L)	135 (135‐145)
Lactate dehydrogenase (U/L)	**750** (120‐246)
AST (U/L)	**210** (13‐40)
ALT (U/L)	**71** (7‐46)
Total bilirubin (mg/dL)	0.25 (0.30‐1.20)
γGT (U/L)	**111** (<38)
ALP (U/L)	**183** (46‐116)
CRP (mg/dL)	**28.51** (<0.5)
Albumin (mg/dL)	**2.78** (3.2‐4.8)
Serum protein electrophoresis	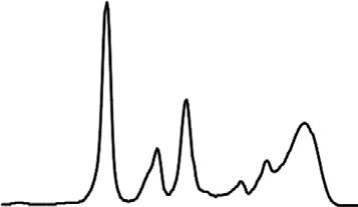
Imunoglobulin G (mg/dL)	**2410** (650‐1690)
ß2 microglobulin (ng/mL)	**8210** (1090‐2530)
Interleukin‐ 6 (pg/L)	**97.2** (<7)
Triglycerides (mg/dL)	**164** (<150)
HDL (mg/dL)	**24.6** (>45.0)
sIL‐2r (pg/mL)	**2752** (458‐1677)
NK cell activity	normal
24‐h urine protein test (mg)	**377** (<150)

In bold: outside reference intervals.

Abbreviations: ALP, Alkaline phosphatase; ALT, Alanine aminotransferase; AST, Aspartate aminotransferase; CRP, C‐reactive protein; HDL, High‐density lipoprotein; NK, Natural killer; sIL‐2r, Soluble interleukin‐2 receptor; γGT, Gamma glutamyl transferase.

**FIGURE 2:**
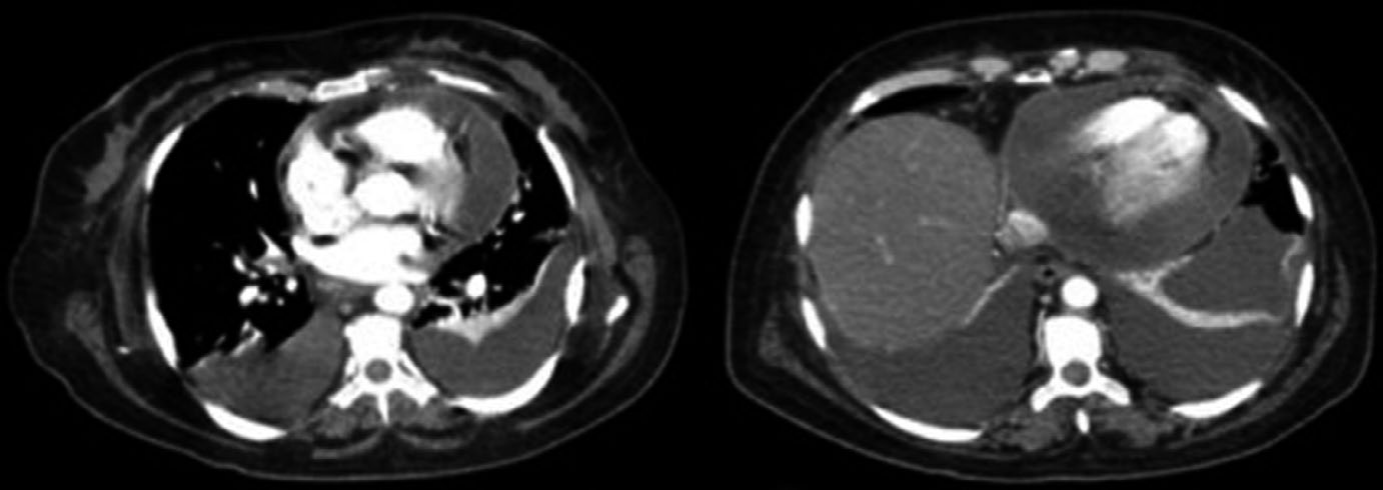
CT scan showing bilateral mediastinal lymphadenopathies and pericardial and bilateral pleural effusion

**FIGURE 3:**
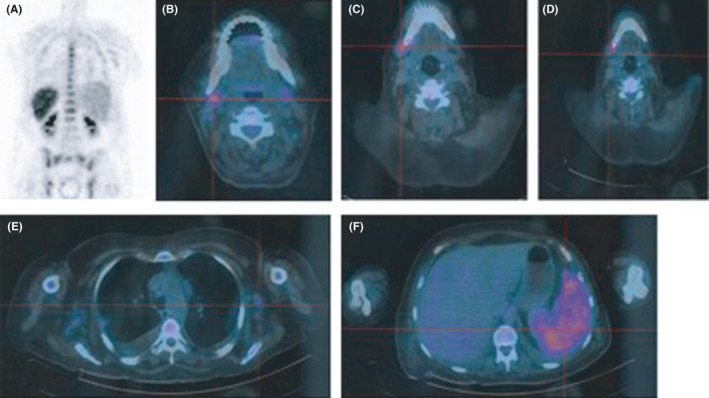
PET scan showing diffuse bone marrow (A), lymphadenopathies—retromandibular (B), submandibular (C), cervical (D), axillar (E), and spleen (F) increased uptake. (SUVmax: 5.64 at right cervical adenopathies)

A neck/chest/abdomen/pelvis computed tomography (CT) revealed bilateral axillar, cervical, mediastinal, and mesenteric lymphadenopathies; splenomegaly and pericardial, peritoneal, and bilateral pleural effusion (Figure 2 **)**; positron emission tomography (PET) scan showed increased diffuse bone marrow, cervical adenopathies (SUVmax: 5.64), and spleen uptake (Figure 3).

Axillar adenopathy, bone and skin biopsies were performed, showing only reactive changes; bone marrow hemophagocytosis evaluation was negative. Ophthalmologic observation suggested *retinitis pigmentosa*.

The study performed allowed the exclusion of liver and heart disease, malignancy, infectious, and autoimmune diseases, as well as genetic defects—HLH Panel by next‐generation sequencing and karyotype (Table 2).

**TABLE 2:** Other diagnostic tests

Parameters	Result
Imunophenotipic study (blood + bone marrow)	Normal phenotype of B,T and NK cells.
Autoimmunity panel	Normal
Adrenal function	Normal
PNH clone	Negative
Mucopolyssacaridases study	Negative
Genetic study (familial HLH)	Negative
Blood cultures	Negatives
*B henselae, B burgodorferi, Brucella spp., C burnetti, C pneumoniae,* CMV, EBV, *H capsulatum*, HHV‐8, HSV, *Leishmania spp., Leptospira spp*.*, M pneumoniae, R conorii, Rubeola, Syphilis, Toxoplasma, VHB, VHC, VIH* (blood**)** *Adenovirus, C parvum, Campylobacter, Rotavirus, Salmonella, Shigella, Y enterocolitica* (heces)	Negatives
Interferon‐γ	Negative
Urine culture	Negative
Duodenal biopsy	No *Whipple* evidence
Bronchial aspirate	Negative
Myeloculture (included TB)	Negative
Caryotype	Normal

Abbreviations: CMV, Cytomegalovirus; EBV, Epstein‐barr virus; HHV‐8, Human herpesvirus‐8; HLH, Hemophagocytic lymphohistiocytosis; HSV, Herpes simplex virus; PNH, Paroxysmal nocturnal hemoglobinuria; TB, tuberculosis.

The patient had a long hospital stay and recurrent deterioration episodes with hypotension, high fever, and other organic dysfunctions. She was admitted to intensive care unit. Although fulfilling only 4 criteria for diagnosis of HLH (*HLH‐2004*), the patient presented a *HScore* of 185 points (indicating a 70%‐80% probability of HLH), and treatment with dexamethasone and cyclosporine was initiated. Clinical stability was achieved, with gradual improvement of initial symptoms, specifically of anemia and hyperferritinemia.

After discharge, there was no adherence to therapeutic prescription with clinical relapse resulting in hospital readmission.

The patient remains under hematology follow‐up, although she is not eligible for hematopoietic stem cell transplantation (HSCT) due to poor family support and no identified underlying genetic defect.

## 3 DISCUSSION

Two diagnostic challenges are relevant in context of HLH. First, a high degree of suspicion is required because of variable clinical presentations, lack of specificity of clinical and laboratory findings, and diversity of causes; prompt recognition of HLH syndrome and diagnosis of underlying cause are critical to enable urgent and appropriate treatment.^6^ Second, it is important to distinguish familial HLH (FHL) from secondary HLH, because HSCT is recommended in FHL.^3^ Furthermore, in the case presented, despite exhaustive study, no etiology for HLH was identified, including infectious, malignancy, autoimmune, or genetic causes. Exclusion of FHL was also important due to limited number of reports of adult‐onset of HLH primary form. Despite greater recognition of the disease, secondary idiopathic forms of HLH are still reported.^4^, ^6^, ^7^, ^8^, ^9^, ^10^, ^11^, ^12^

The presence of dysmorphic face, short stature, mild cognitive impairment, and blindness could suggest *Laurence‐Moon‐Bardet‐Biedl syndrome*, but genetic testing was not performed. No association between this hereditary disease and HLH was previously reported in literature.

Differential diagnostic workup should not delay initiation of HLH‐specific treatment in those who are acutely ill. Indeed, adults with HLH have poor outcomes even with aggressive treatment.^3^ In the case presented, once treatment was started, clinical stability was achieved, with clinical and analytical improvement—gradual improvement of initial symptoms and hemoglobin levels and decreased of ferritin, CRP, sedimentation velocity, and LDH levels.

Due to the absence of most characteristic and common features present in pediatric literature reports, HScore was used in this case to guide diagnosis, but above all, clinical judgment and a high index of suspicion were required. Features of HLH in adults are still in debate, and there are no established criteria for this population, so current diagnostic criteria should be used with caution.

Frequently, diagnosis of HLH is made for patients who only partly meet the most stringent criteria, and this presumptive diagnosis depends on a careful consideration of the presence or absence of specific elements embedded in diagnostic criteria, results of additional laboratory tests, and a nuanced view of overall clinical status. This emphasizes that clinical judgment should be encouraged instead of relying solely on strict adherence to pre‐established diagnostic criteria, especially in adults.

In this case, hemophagocytosis was not observed in bone marrow biopsy, but it is neither pathognomonic of nor required for the diagnosis of HLH.^3^, ^7^, ^8^, ^10^, ^12^ Significant cytopenias and disturbance of NK cell activity were also absent,^6^, ^7^, ^8^, ^10^, ^11^ and there were nonconcordant fibrinogen levels.^4^, ^7^, ^8^, ^10^, ^12^, ^13^, ^14^, ^15^ In contrast, there were some features suggesting HLH diagnosis in adult patients: hyperferritinemia^4^, ^15^; fever^4^, ^6^, ^7^, ^8^, ^9^, ^11^, ^12^, ^13^, ^15^; splenomegaly^4^, ^6^, ^7^, ^8^, ^9^, ^11^, ^12^, ^13^, ^14^; abnormal liver enzymes^4^, ^15^; high LDH^4^, ^6^, ^7^, ^8^, ^9^, ^10^, ^11^, ^12^; presence of other inflammatory parameters (increased sedimentation velocity,^9^, ^11^ CRP,^4^, ^6^, ^9^, ^10^, ^11^, ^12^ haptoglobin, and alpha globulins; unusual leukocytosis,^12^, ^15^ thrombocytosis,^9^ and hypergammaglobulinemia); enlarged lymph nodes^6^, ^9^, ^10^, ^11^, ^12^, ^14^ (mainly cervical and mediastinal); serous effusions^4^, ^9^, ^12^, ^14^, ^15^; hypoalbuminemia^6^, ^7^, ^8^; renal impairment parameters^9^, ^11^; skin rash^14^; multiorgan failure mimicking septic shock^2^, ^10^, ^14^, ^15^; raised D‐dimer levels^15^; and low HDL cholesterol levels.

Mild elevation in leukocytes probably reflects balanced effects of cytokines (eg: IL‐6 and IFN‐γ), different release patterns, and concurrent effects on inflammatory markers of each.

We present a clinical case of a secondary idiopathic HLH in an adult patient, first appearing with unspecific complaints, followed by sepsis‐like evolution and requiring advanced life support. Clinical judgment and a high index of suspicion are required. This report intends to contribute to the framing of clinical recommendations for this rare, heterogeneous, and complex clinical entity.

## CONFLICT OF INTEREST

None declared.

## AUTHOR CONTRIBUTIONS

All authors: conceived, wrote, and reviewed the manuscript. Rabadão T, Eulálio M, and Silva F: actively involved in the clinical care of the patient.

## Data Availability

Data sharing is not applicable to this article as no new data were created on analyzed in this study.

## References

[1] La Roséee P, Homme A, Hines M, et al. Recommendations for the management of hemophagocytic lymphohistiocytosis in adults. Blood. 2019;133(23):2465‐2477. 30992265 10.1182/blood.2018894618

[2] Jordan MB, Allen CE, Greenberg J, et al. Challenges in the diagnosis of hemophagocytic lymphohistiocytosis: recommendations from the North American Consortium for Histiocytosis. Ped Blood Cancer. 2019;66(11):e27929. 10.1002/pbc.27929 PMC7340087 31339233

[3] Al‐Samkari H, Berliner N. Hemophagocytic Lymphohistiocytosis. Annu Rev Pathol Mech Dis. 2018;13:27‐49. 10.1146/annurev-pathol-020117-043625 28934563

[4] Zhang Z, Wang J, Ji B, et al. Clinical presentation of hemophagocytic lymphohistiocytosis in adults is less typical than in children. Clinic. 2016;71(4):205‐209. 10.6061/clinics/2016(04)05 PMC4825195 27166770

[5] Mehta P, McAuley DF, Brown M, et al. COVID‐19: consider cytokine storm syndromes and immunosuppression. Lancet. 2020;395(10229):1033‐1034. 32192578 10.1016/S0140-6736(20)30628-0 PMC7270045

[6] Birndt S, Schenk T, Heinevetter B, et al. Hemophagocytic lymphohistiocytosis in adults: collaborative analysis of 137 cases of a nationwide German registry. J Cancer Res Clin Oncol. 2020;146:1065‐1077. 32076823 10.1007/s00432-020-03139-4 PMC7085479

[7] Lim SH, Park S, Jang JH, et al. Clinical significance of bone marrow hemophagocytosis in adult patients with malignancy and non‐malignancy‐induced hemophagocytic lymphohistiocytosis. Ann Hematol. 2016;95(2):325‐335. 26453074 10.1007/s00277-015-2523-8

[8] Otrock ZK, Eby CS. Clinical characteristics, prognostic factors and outcomes of adult patients with hemophagocytic lymphohistiocytosis. Am J Hem. 2015;90(3):220‐224. 10.1002/ajh.23911 25469675

[9] Diack ND, Kane BS, Fall S, et al. Adult Hemophagocytic lymphohistiocytosis in Sub‐Saharan Area: a retrospective study of 26 cases. Cureus. 2020;12(3):e7258. 32195069 10.7759/cureus.7258 PMC7075479

[10] Rivière S, Galicier L, Coppo P, et al. Reactive Hemophagocytic syndrome in adults: a retrospective analysis of 162 patients. Am J Med. 2014;127(11):1118‐1125. 24835040 10.1016/j.amjmed.2014.04.034

[11] Li J, Wang Q, Zheng W, et al. Hemophagocytic Lymphohistiocytosis– Clinical Analysis of 103 Adult Patients. Medicine. 2014;93(2):100‐105. 24646466 10.1097/MD.0000000000000022 PMC4616310

[12] Fatima Z, Khan A, Tariq U, Sohail MS. Hemophagocytic lymphohistiocytosis: a case series. Cureus. 2018;10(4):e2545. 29963339 10.7759/cureus.2545 PMC6021184

[13] Gars E, Purington N, Scott G, et al. Bone marrow histomorphological criteria can accurately diagnose hemophagocytic lymphohistiocytosis. Haematologica. 2018;103(10):1635‐1641. 29903767 10.3324/haematol.2017.186627 PMC6165820

[14] Rajagopala S, Singh N, Agarwal R, Gupta D, Das R. Severe hemophagocytic lymphohistiocytosis in adults‐experience from an intensive care unit from North India. Indian J Crit Care Med. 2012;16(4):198‐203. 23559726 10.4103/0972-5229.106501 PMC3610451

[15] Kay CL, Rendo MJ, Gonzales P, Beganovic SG, Czader M. Successful modified therapy in a patient with probable infection‐associated Hemophagocytic lymphohistiocytosis. Case Rep Oncol Med. 2019;2019:9781065. 31583147 10.1155/2019/9781065 PMC6754915

